# Steroidomic Changes in the Cerebrospinal Fluid of Women with Multiple Sclerosis

**DOI:** 10.3390/ijms26125904

**Published:** 2025-06-19

**Authors:** Radmila Kancheva, Eva Kubala Havrdová, Marta Velíková, Ludmila Kancheva, Josef Včelák, Radek Ampapa, Michal Židó, Ivana Štětkářová, Tereza Škodová, Martin Hill

**Affiliations:** 1 Institute of Endocrinology, 11000 Prague, Czech Republic; mvelikova@endo.cz (M.V.); lkancheva@endo.cz (L.K.); jvcelak@endo.cz (J.V.); tskodova@endo.cz (T.Š.); 2 Department of Neurology, First Faculty of Medicine, Charles University, 12808 Prague, Czech Republic; ehavr@lf1.cuni.cz; 3 MS Center, Jihlava Hospital, 58633 Jihlava, Czech Republic; ampapar@gmail.com; 4 Department of Neurology 3FM CU and UHKV, Third Faculty of Medicine, Charles University, 12808 Prague, Czech Republic; michal.zido@fnkv.cz (M.Ž.); ivana.stetkarova@fnkv.cz (I.Š.)

**Keywords:** multiple sclerosis, cerebrospinal fluid, steroidomics, GC-MS/MS, multivariate statistics

## Abstract

Multiple sclerosis (MS) is a long-term disease that causes inflammation and damage to the nervous system. This study evaluated steroidomic alterations related to MS in 57 female MS patients during the follicular phase and 17 during the luteal phase, as well as in age- and phase-matched controls. The data showed that (1) unconjugated and conjugated steroids were strongly linked between the blood and CSF. (2) MS patients have lower levels of unconjugated steroids compared to controls. However, unchanged levels of conjugated steroids suggest a possible increase in steroid sulfotransferase functioning. (3) MS patients show altered levels of steroids linked to 11β-hydroxylase (CYP11B1) function. While direct enzyme activity was not measured, disrupted cortisol biosynthesis—potentially linked to reduced functioning of both CYP11B1 and 17α-hydroxylase/17,20-lyase—is associated with more severe cases of MS. (4) Reduced levels of 5α/β-steroids and protective GABAergic 3α-hydroxy-5α/β-steroids in MS patients might be linked to the pathophysiology of MS. (5) A potential increase in AKR1C3 function in MS could contribute to inflammation, as this enzyme catalyzes the synthesis of both steroids and prostaglandins. However, direct measurements of enzyme activity are needed to confirm this hypothesis. (6) Lower pregnenolone levels in MS patients might weaken neuroprotection, while higher pregnenolone sulfate levels could support cognitive function. (7) Lower levels of protective pregnenolone, DHEA, and androstenediol were associated with worse MS, suggesting these steroids may help shield against the disease.

## 1. Introduction

Multiple sclerosis (MS) is a chronic inflammatory disease of the central nervous system (CNS) associated with demyelination and neurodegeneration that is more common in women [1]. MS is accompanied by varying degrees of inflammation associated with the secretion of autoantigens, demonstrating an autoimmune inflammatory response. Inflammation and neurodegeneration go together from the beginning of the disease [2].

### 1.1. Origin and Role of Steroids in the Brain

Steroids found in the brain derive from two primary sources. Neurosteroids are synthesized directly within the brain from cholesterol. These locally synthesized steroids are essential for regulating neuronal excitability, brain plasticity, and behavior. Peripheral steroids, synthesized in peripheral tissues, primarily in endocrine glands, can enter the CNS through the bloodstream, crossing the blood–brain barrier and the blood–cerebrospinal fluid barrier. Once in the brain, these steroids may undergo further metabolism to become active neurosteroids [3,4].

Steroids like glucocorticoids and neurosteroids protect neurons from damage caused by oxidative stress, inflammation, and excitotoxicity. They regulate the formation of new neurons, particularly in the hippocampus, which is crucial for memory, learning, mood, anxiety, and other behavioral processes. Steroids also influence synaptic plasticity, which is essential for cognitive functions. In addition, glucocorticoids mediate the brain’s response to stress. While the 5α/β-reduced GABAergic steroids, characterized by a hydroxyl group at the 3α position, such as allopregnanolone, pregnanolone, androsterone, and androstanediol, are neuroprotective, similar to the short-term anti-inflammatory effects of cortisol at appropriate concentrations, their long-term effects on brain health remain unclear. However, excessive glucocorticoids, particularly in cases of chronic elevation, are undesirable as they can cause neuronal damage and lead to mood disorders, such as depression and anxiety [5,6].

Various physiological and pathological conditions can lead to an increase in neuroprotective steroids within the CNS. Acute stress can trigger the production of neurosteroids, such as allopregnanolone, which modulate the GABAergic system and provide neuroprotective effects. Brain injuries, including ischemic events and traumatic brain injuries, can stimulate neurosteroid synthesis as part of the brain’s protective response to damage. Hormonal fluctuations, such as those occurring during pregnancy or the menstrual cycle, can affect neurosteroid levels in the CNS. Medications, such as synthetic neurosteroid analogs or agents that boost neurosteroid synthesis, can increase their concentrations in the brain [7,8,9].

The rise of undesirable steroids in the CNS can be caused by multiple factors. Chronic stress resulting in prolonged activation of the hypothalamic–pituitary–adrenal axis causes persistently elevated cortisol levels, potentially leading to neurotoxic effects on the brain, especially in the hippocampus. Also, disorders like Cushing’s syndrome, marked by the overproduction of cortisol, can lead to increased glucocorticoid levels in the central nervous system. Prolonged or high-dose administration of synthetic corticosteroids may cause adverse effects on the CNS, e.g., cognitive impairments and mood disorders. Certain infections or inflammatory conditions affecting the CNS can disrupt steroid metabolism, resulting in an imbalance and increased levels of harmful steroids. Disruptions to the normal pattern of cortisol secretion, such as those resulting from shift work or sleep disorders, can lead to increased glucocorticoid levels [10,11].

### 1.2. Multiple Sclerosis and Steroids

Sex hormones such as testosterone and progesterone are anti-inflammatory, while estradiol has a bipotential effect, depending on its concentration [12]. Furthermore, changes in sex hormone levels before the menstrual cycle are associated with an aggravation of MS [13]. In addition, the incidence of MS symptoms decreases in the last three months of pregnancy and increases again after delivery. These changes are also linked to immunological and hormonal alterations [14].

#### 1.2.1. Δ^5^ Steroids

Pregnenolone, dehydroepiandrosterone (DHEA) and their sulfates penetrate the blood–brain barrier (BBB) and their therapeutic administration affects their concentrations in the CNS [15,16]. Pregnenolone, DHEA and dehydroepiandrosterone sulfate (DHEAS) are neuroprotective [17]. Pregnenolone sulfate (PregS) and DHEAS are neuroactive steroids (NAS) that modulate several types of ionotropic receptors and may alleviate some of the adverse effects accompanying MS (see [18]). The neuroprotective effects of DHEA/DHEAS can also be ascribed to their modulatory effect on type A γ-aminobutyric acid receptors (GABA_A_R).

#### 1.2.2. Corticoids

Cortisol is known to increase the body’s readiness in stressful situations by increasing glucose levels via gluconeogenesis [19]. Cortisol affects the activity of several neurotransmitter systems that influence reward processing, attention regulation, executive function, mood and emotion and also suppresses the synthesis, release and metabolism of serotonin, which increases the risk of depression. Moreover, chronically increased cortisol secretion may result in cognitive impairment [19].

#### 1.2.3. GABAergic Steroids

The GABAergic steroids as allopregnanolone exert various neuroprotective effects, e.g., alleviation of neurobehavioral deficits and counter regulation of neuropathology and inflammation [20]. Recent studies have shown the inhibition of neuroinflammation by allopregnanolone via activation of toll-like receptor 4 protein in macrophages and in the brain [21].

## 2. Results

### 2.1. Correlations Between Steroids in Serum and Cerebrospinal Fluid

The correlations between steroid levels in serum and CSF are shown in Table 1. Their effect size ranged from medium to very large.

### 2.2. Alterations in Steroid Levels

Steroidomic changes in CSF in female patients with MS compared to controls, taking into account the phase of the menstrual cycle and the age of the volunteers, are presented in Table 2. The differentiation between female patients and controls based on steroidomic changes using OPLS models is also shown in Supplementary Tables S1 and S2 and in Supplementary Figures S1 and S2 for the follicular and luteal phases of the menstrual cycle, respectively. The analysis of steroid levels in patients showed the following trends:

Unconjugated steroids: A strong trend towards lower levels was found (n = 12 lower, n = 6 unchanged, n = 1 higher, *p* = 0.002).

Conjugated steroids: No significant trend was observed, with similar numbers being lower (n = 6), unchanged (n = 9), or higher (n = 4) (*p* = 0.541).

#### 2.2.1. Δ^5^ and Δ^4^ Steroids

The comparison of Δ^5^ and Δ^4^ steroid levels in patients revealed no significant overall trend related to MS (*p* = 0.138). Among Δ^5^ steroids, lower levels were observed for pregnenolone, 16α-hydroxypregnenolone, androstenediol, and 5-androstene-3β,16α,17β-triol sulfate, while higher levels were noted for pregnenolone sulfate, androstenediol sulfate, and 7α-hydroxy-DHEA. For Δ^4^ steroids, all measured levels (17,20α-dihydroxy-4-pregnene-3-one, 16α-hydroxyprogesterone, androstenedione, and 11β-hydroxyandrosterone) were lower in patients, suggesting a borderline trend (*p* = 0.072), possibly due to the limited number of steroids analyzed.

#### 2.2.2. 11β-Hydroxy-Androstanes (C19 Δ^4^ and 5α/β Steroids)

Among 11β-hydroxy-androstanes, most had significantly lower levels in MS patients (n = 4), with one showing no change (n = 1) and none showing higher levels (n = 0). This suggested a borderline trend toward lower levels in MS patients (*p* = 0.063), though it did not reach significance, likely due to the small number of steroids analyzed.

#### 2.2.3. GABAergic Steroids

Steroids associated with GABAergic effects, including 5α/β-reduced steroids and specifically 3α-hydroxy-5α/β-steroids, were analyzed. For 5α/β-reduced steroids, most had significantly lower levels in patients (n = 10), while fewer showed no change (n = 5) or higher levels (n = 1), indicating a significant trend towards lower levels (*p* = 0.022). Similarly, for 3α-hydroxy-5α/β-steroids, more had lower levels in patients (n = 8), with fewer showing no change (n = 2) or higher levels (n = 2), also showing a significant trend towards lower levels (*p* = 0.021).

#### 2.2.4. 17-oxo- and 17 β-Hydroxy-Androstanes

For 17-oxo-androstanes, most showed significantly lower levels in patients (n = 9), with fewer showing no change (n = 5) or higher levels (n = 1), indicating a significant trend towards lower levels (*p* = 0.012). In contrast, for 17β-hydroxy-androstanes, levels were evenly distributed between lower (n = 2), unchanged (n = 4), and higher (n = 4) values, showing no significant trend (*p* = 1).

### 2.3. Correlation Between Indices of MS Severity and Steroids

The relationships between MS severity and steroid levels were analyzed separately for the follicular (FP) and luteal (LP) phases of the menstrual cycle, as shown in Table 3, Table 4, Table 5, Table 6, Table 7, Table 8, Table 9 and Table 10.

#### 2.3.1. Expanded Disability Status Scale (EDSS)

Table 3 shows that in the follicular phase (FP), both pregnane and androstane steroids have negative correlations with EDSS. In the LP, androstane steroids still show negative correlations, but pregnane steroids have positive correlations with EDSS (Table 4).

**Table 3:** Relationships between Expanded Disability Status Scale (EDSS) and explaining variables for follicular phase as evaluated by models of orthogonal predictions to latent structure (OPLS) and ordinary multiple regression (OMR). Corresponding diagnostic outputs are shown in Supplementary Figure S3.

	Variable	OPLS, Predictive Component								Multiple Regression	
	
		Variable Importance	t-Statistic		Component Loading	t-Statistic	R			t-Statistic	
Explaining Variables	Pregnenolone	1.35	4.77	**	−0.304	−4.50	−0.640	**		−2.64	*
	17-Hydroxypregnenolone	1.075	2.16	*	−0.310	−3.29	−0.654	**		−2.09	*
	7α-Hydroxy-DHEA	0.667	2.43	*	−0.379	−8.87	−0.798	**		−1.78	
	5-Androstene-3β,7α,17β-triol	0.79	3.18	**	−0.380	−6.39	−0.800	**		−1.88	
	5-Androstene-3β,7β,17β-triol	0.93	2.91	*	−0.366	−8.15	−0.772	**		−1.67	
	17,20α-Dihydroxy-4-pregnene-3-one	0.889	2.04	*	−0.316	−7.71	−0.667	**		−1.69	
	Androstenedione	1.317	4.40	**	−0.371	−7.08	−0.783	**		−5.34	**
	5β-Pregnane-3α,17,20α-triol	0.73	3.70	**	−0.334	−7.22	−0.703	**		−1.92	*
	Androsterone	1.093	1.94	*	−0.210	−3.15	−0.443	**		−1.60	
	11β-Hydroxyandrostenedione	0.915	3.13	**	−0.308	−13.16	−0.649	**		−2.95	*
Explained Variable	Expanded Disability Status Scale				1.000	2.52	0.388	*			
	Follicular phase	R^2^ = 15.1%, Q^2^ = 8.4%, CV-ANOVA: F = 2.03, *p* = 0.144									

R = Component loading expressed as a correlation coefficient with predictive component, * *p* < 0.05, ** *p* < 0.01, R^2^ = Explained variance, Q^2^ = Predictive ability, CV-ANOVA = cross-validated ANOVA, F = F-statistic, *p* = statistical significance.

**Table 4:** Relationships between Expanded Disability Status Scale (EDSS) and explaining variables for luteal phase as evaluated by models of orthogonal predictions to latent structure (OPLS) and ordinary multiple regression (OMR). Corresponding diagnostic outputs are shown in Supplementary Figure S4.

	Variable	OPLS, Predictive Component								Multiple Regression	
	
		Variable Importance	t-Statistic		Component Loading	t-Statistic	R			t-Statistic	
Explaining Variables	Dehydroepiandrosterone	0.645	2.24	*	0.145	1.06	0.180			1.58	
	5α,20α-Tetrahydroprogesterone	0.816	2.26	*	0.458	2.83	0.566	*		1.38	
	5β-Pregnane-3α,20α-diol	1.053	3.91	**	0.455	3.41	0.562	**		1.93	*
	5β-Androstane-3α,17β-diol	1.092	2.73	*	−0.574	−2.33	−0.709	*		−1.98	*
	11β-Hydroxyandrostenedione	1.271	3.33	**	−0.521	−1.87	−0.644			−2.35	*
Explained Variable	Expanded Disability Status Scale				1.000	5.09	0.710	**			
	Luteal phase	R^2^ = 50.3%, Q^2^ = 24.4%, CV-ANOVA: F = 1.93, *p* = 0.187									

R = Component loading expressed as a correlation coefficient with predictive component, * *p* < 0.05, ** *p* < 0.01, R^2^ = Explained variance, Q^2^ = Predictive ability, CV-ANOVA = cross-validated ANOVA, F = F-statistic, *p* = statistical significance.

#### 2.3.2. Timed 25-Foot Walk (T25-FW)

For the T25-FW, the correlations in the FP are complex (Table 5). The Δ^5^ and Δ^4^ pathway steroids show negative correlations, while sulfates of 5α/β-steroids and unconjugated 11β-hydroxyepiandrosterone show positive correlations. In the LP, some androstane steroids and the pregnanes 16α-hydroxyprogesterone and 5α,20α-tetrahydroprogesterone correlate with T25-FW (Table 6).

**Table 5:** Relationships between Timed 25-Foot Walk (T25-FW) and explaining variables for follicular phase as evaluated by models of orthogonal predictions to latent structure (OPLS) and ordinary multiple regression (OMR). Corresponding diagnostic outputs are shown in Supplementary Figure S5.

	Variable	OPLS, Predictive Component								Multiple Regression	
	
		Variable Importance	t-Statistic		Component Loading	t-Statistic	R			t-Statistic	
Explaining Variables	Age	0.915	2.27	*	0.146	1.71	0.342			1.99	*
	Pregnenolone	0.671	2.62	*	−0.100	−0.96	−0.233			−2.22	*
	17-Hydroxypregnenolone	0.873	2.98	*	−0.214	−4.58	−0.502	**		−2.39	*
	16α-Hydroxypregnenolone	1.171	3.83	**	−0.200	−3.66	−0.469	**		−2.57	*
	Dehydroepiandrosterone	0.67	2.71	*	−0.167	−2.59	−0.390	*		−1.94	*
	16α-Hydroxyprogesterone	0.615	2.11	*	−0.148	−1.59	−0.345			−2.19	*
	Allopregnanolone, C	1.517	4.96	**	0.310	5.45	0.726	**		4.75	**
	Isopregnanolone, C	1.587	5.08	**	0.321	4.86	0.751	**		3.57	**
	Pregnanolone, C	1.241	6.39	**	0.339	8.33	0.793	**		4.93	**
	5β-Pregnane-3α,20α-diol, C	0.682	2.63	*	0.225	1.96	0.528	*		2.91	*
	Androsterone, C	0.825	3.24	**	0.273	4.16	0.638	**		3.87	**
	Epiandrosterone, C	1.167	4.22	**	0.278	6.34	0.651	**		2.78	*
	Etiocholanolone, C	0.804	2.42	*	0.278	5.23	0.650	**		3.17	**
	Epietiocholanolone, C	1.047	3.28	**	0.339	5.91	0.793	**		3.45	**
	5α-Androstane-3β,17β-diol, C	0.956	5.92	**	0.287	9.13	0.671	**		5.36	**
	11β-Hydroxyandrosterone	0.674	2.68	*	0.125	1.81	0.294			1.90	*
	11β-Hydroxyandrosterone, C	1.057	5.26	**	0.242	6.55	0.566	**		3.00	**
	11β-Hydroxyepiandrosterone	0.812	2.29	*	0.150	1.81	0.351			1.92	*
Explained Variable	Timed 25-Foot Walk				1.000	11.44	0.726	**			
	Follicular phase	R^2^ = 46.8%, Q^2^ = 28.8%, CV-ANOVA: F = 11.7, *p* < 0.001									

R = Component loading expressed as a correlation coefficient with predictive component, * *p* < 0.05, ** *p* < 0.01, R^2^ = Explained variance, Q^2^ = Predictive ability, CV-ANOVA = cross-validated ANOVA, F = F-statistic, *p* = statistical significance, C = conjugated steroid.

**Table 6:** Relationships between Timed 25-Foot Walk (T25-FW) and explaining variables for luteal phase as evaluated by models of orthogonal predictions to latent structure (OPLS) and ordinary multiple regression (OMR). Corresponding diagnostic outputs are shown in Supplementary Figure S6.

	Variable	OPLS, Predictive Component								Multiple Regression	
	
		Variable Importance	t-Statistic		Component Loading	t-Statistic	R			t-Statistic	
Explaining Variables	Age	1.162	1.98	*	−0.233	−0.80	−0.391			−1.69	
	Androstenediol	1.117	3.35	**	−0.362	−2.31	−0.609	*		−2.98	*
	5-Androstene-3β,7α,17β-triol	0.899	2.83	*	−0.412	−1.90	−0.693	*		−4.13	**
	5-Androstene-3β,7β,17β-triol	0.828	2.08	*	−0.374	−1.49	−0.629			−3.08	**
	16α-Hydroxyprogesterone	0.932	6.41	**	−0.388	−3.14	−0.652	**		−2.74	*
	Androstenedione	1.021	1.95	*	−0.401	−3.63	−0.675	**		−1.66	
	5α,20α-Tetrahydroprogesterone	0.955	2.48	*	−0.237	−1.27	−0.398			−1.90	*
	11β-Hydroxyandrostenedione	1.096	3.86	**	−0.349	−3.87	−0.586	**		−2.93	*
	11β-Hydroxyandrosterone	0.941	2.97	*	−0.282	−1.13	−0.475			−2.06	*
Explained Variable	Timed 25-Foot Walk				1.000	8.57	0.783	**			
	Luteal phase	R^2^ = 61.3% Q^2^ = 49.8%, CV-ANOVA: F = 4.97, *p* = 0.031									

R = Component loading expressed as a correlation coefficient with predictive component, * *p* < 0.05, ** *p* < 0.01, R^2^ = Explained variance, Q^2^ = Predictive ability, CV-ANOVA = cross-validated ANOVA, F = F-statistic, *p* = statistical significance.

#### 2.3.3. 9-Hole Peg Test (9-HPT) for MS, Right Hand

In the FP, the T25-FW (right hand) showed negative correlations with pregnenolone, its 16α-hydroxy metabolite, DHEA, and androsterone (Table 7). In the LP, negative correlations were found with 17-hydroxypregnenolone sulfate, 16α-hydroxypregnenolone, DHEA, its sulfate, and several 5α/β-metabolites of DHEA. Conversely, androstenedione and 5α,20α-tetrahydroprogesterone showed positive correlations in the LP (Table 8).

**Table 7:** Relationships between 9-Hole Peg Test (9-HPT), right hand and explaining variables for follicular phase as evaluated by models of orthogonal predictions to latent structure (OPLS) and ordinary multiple regression (OMR). Corresponding diagnostic outputs are shown in Supplementary Figure S7.

		OPLS, Predictive Component								Multiple Regression	
		
	Variable	Variable Importance	t-Statistic		Component Loading	t-Statistic	R			t-Statistic	
Explaining Variables	Pregnenolone	1.115	5.77	**	−0.496	−5.14	−0.697	**		−2.76	*
	16α-Hydroxypregnenolone	1.130	4.70	**	−0.581	−10.20	−0.817	**		−3.55	**
	Dehydroepiandrosterone	0.948	2.61	*	−0.507	−7.70	−0.713	**		−2.67	*
	Androsterone	0.763	3.21	**	−0.407	−4.22	−0.572	**		−3.62	**
Explained Variable	9-Hole Peg Test, right hand				1.000	2.01	0.517	*			
	Follicular phase	R^2^ = 26.7%, Q^2^ = 21.2%, CV-ANOVA: F = 3.64, *p* = 0.040									

R = Component loading expressed as a correlation coefficient with predictive component, * *p* < 0.05, ** *p* < 0.01, R2 = Explained variance, Q^2^ = Predictive ability, CV-ANOVA = cross-validated ANOVA, F = F-statistic, *p* = statistical significance.

**Table 8:** Relationships between 9-Hole Peg Test (9-HPT), right hand and explaining variables for luteal phase as evaluated by models of orthogonal predictions to latent structure (OPLS) and ordinary multiple regression (OMR). Corresponding diagnostic outputs are shown in Supplementary Figure S8.

	Variable	OPLS, Predictive Component								Multiple Regression	
	
		Variable Importance	t-Statistic		Component Loading	t-Statistic	R			t-Statistic	
Explaining Variables	17-Hydroxypregnenolone, C	0.944	3.78	**	−0.265	−4.36	−0.426	**		−0.58	
	16α-Hydroxypregnenolone	0.474	2.07	*	−0.102	−0.98	−0.164			1.91	*
	Dehydroepiandrosterone	0.648	2.00	*	−0.198	−1.46	−0.318			0.35	
	Dehydroepiandrosterone, C	1.029	5.95	**	−0.290	−4.28	−0.466	**		−2.04	*
	5-Androstene-3β,16α,17β-triol, C	0.963	3.22	**	−0.269	−2.30	−0.432	*		−1.63	
	Androstenedione	0.741	2.06	*	0.241	1.91	0.387	*		2.62	*
	5α,20α-Tetrahydroprogesterone	1.26	2.49	*	0.380	2.47	0.611	*		1.41	
	Androsterone, C	0.933	2.82	*	−0.288	−3.70	−0.463	**		−0.62	
	Etiocholanolone, C	0.855	3.97	**	−0.233	−4.46	−0.374	**		−0.80	
	Epietiocholanolone, C	0.734	2.24	*	−0.271	−1.87	−0.435			0.19	
	5α-Androstane-3β,17β-diol, C	1.206	5.99	**	−0.361	−2.84	−0.579	*		−1.81	
	11β-Hydroxyandrosterone, C	1.657	12.51	**	−0.435	−5.35	−0.698	**		−1.99	*
Explained Variable	9-Hole Peg Test, right hand				1.000	3.78	0.825	**			
	Luteal phase	R^2^ = 68.1%, Q^2^ = 34.8%, CV-ANOVA: F = 1.07, *p* = 0.432									

R = Component loading expressed as a correlation coefficient with predictive component, * *p* < 0.05, ** *p* < 0.01, R2 = Explained variance, Q^2^ = Predictive ability, CV-ANOVA = cross-validated ANOVA, F = F-statistic, *p* = statistical significance, C = conjugated steroid.

#### 2.3.4. 9-Hole Peg Test (9-HPT) for MS, Left Hand

For 9-HPT (left hand) in the FP, there were negative correlations with pregnenolone and some 5α-steroids, except for a positive correlation with 5-androstene-3β,16α,17β-triol sulfate (Table 9). In the LP, 9-HPT (left hand) showed negative correlations with this steroid and with three 11β-hydroxy-androstanes (Table 10).

**Table 9:** Relationships between 9-Hole Peg Test (9-HPT), left hand and explaining variables for follicular phase as evaluated by models of orthogonal predictions to latent structure (OPLS) and ordinary multiple regression (OMR).

	Variable	OPLS, Predictive Component								Multiple Regression	
	
		Variable Importance	t-Statistic		Component Loading	t-Statistic	R			t-Statistic	
Explaining Variables	Pregnenolone	1.267	2.99	*	−0.547	−3.23	−0.733	**		−2.14	*
	5-Androstene-3β,16α,17β-triol, C	1.158	2.49	*	0.469	2.45	0.629	*		2.30	*
	5α,20α-Tetrahydroprogesterone	0.774	2.02	*	−0.320	−1.52	−0.429			−1.05	
	5α-Pregnane-3α,20α-diol, C	0.65	2.01	*	−0.219	−1.76	−0.293			−1.00	
	Androsterone, C	1.067	3.18	**	−0.390	−2.68	−0.523	*		−1.32	
	5α-Androstane-3α,17β-diol, C	0.946	2.66	*	−0.431	−2.87	−0.578	*		−0.89	
Explained Variable	9-Hole Peg Test, left hand				1.000	2.86	0.608	*			
	Follicular phase	R^2^ = 37.0%, Q^2^ = 23.3%, CV-ANOVA: F = 2.12, *p* = 0.104									

R = Component loading expressed as a correlation coefficient with predictive component, * *p* < 0.05, ** *p* < 0.01, R^2^ = Explained variance, Q^2^ = Predictive ability, CV-ANOVA = cross-validated ANOVA, F = F-statistic, *p* = statistical significance, C = conjugated steroid.

**Table 10:** Relationships between 9-Hole Peg Test (9-HPT), left hand and explaining variables for luteal phase as evaluated by models of orthogonal predictions to latent structure (OPLS) and ordinary multiple regression (OMR).

	Variable	OPLS, Predictive Component								Multiple Regression	
	
		Variable Importance	t-Statistic		Component Loading	t-Statistic	R			t-Statistic	
Explaining Variables	5-Androstene-3β,16α,17β-triol, C	1.024	3.22	**	−0.595	−2.27	−0.712	*		−3.29	**
	11β-Hydroxyandrostenedione	0.64	1.95	*	−0.412	−1.67	−0.493			−1.94	*
	11β-Hydroxyandrosterone, C	1.19	3.28	**	−0.466	−1.22	−0.557			−3.45	**
	11β-Hydroxyepiandrosterone	1.061	2.57	*	−0.540	−1.69	−0.646			−2.40	*
Explained Variable	9-Hole Peg Test, left hand				1.000	4.86	0.824	**			
	Luteal phase	R^2^ = 67.9%, Q^2^ = 63.7%, CV-ANOVA: F = 7.90, *p* = 0.010									

R = Component loading expressed as a correlation coefficient with predictive component, * *p* < 0.05, ** *p* < 0.01, R^2^ = Explained variance, Q^2^ = Predictive ability, CV-ANOVA = cross-validated ANOVA, F = F-statistic, *p* = statistical significance, C = conjugated steroid.

## 3. Discussion

### 3.1. Correlations Between Steroids in Serum and Cerebrospinal Fluid

Strong correlations of steroid levels between the circulation and CSF align with previous findings [22], showing that free and sulfated steroids pass through the BBB into the CNS and that circulating steroids play a key role in shaping the CNS steroidome [16].

### 3.2. Alterations in Steroid Levels

The significant trend of lower unconjugated steroid levels suggests reduced steroidogenesis in MS patients compared to controls. Meanwhile, the lack of significance in conjugated steroids may point to increased sulfotransferase (SULT2A1) functioning in MS patients.

#### 3.2.1. Δ^5^ and Δ^4^ Steroids

Although Δ^5^ and Δ^4^ steroids showed no significant trend in relation to MS, the Δ^5^ steroid pregnenolone and all tested Δ^4^ steroids had significantly lower levels in MS patients. Unconjugated pregnenolone is a neuroprotective steroid that counteracts glutamate-induced neurotoxicity, stabilizes microtubules, promotes neurite growth, and supports myelination [17].

In contrast, MS patients had higher levels of glutamatergic pregnenolone sulfate and immunomodulatory androstanes, such as androstenediol sulfate and 7α-hydroxy-DHEA. Pregnenolone sulfate influences various ionotropic receptors and may enhance cognitive function, reduce pain transmission, and alleviate fear [18].

Δ^5^ androstanes and their stronger 7α/β- and 16α-hydroxy-metabolites help reduce the severity of autoimmune diseases [23,24,25,26,27,28]. Interestingly, autoimmune diseases may lower adrenal Δ^5^ androgen production [23]. These steroids regulate the balance between Th1 and Th2 cells, either promoting Th1 or suppressing both types [27,29]. Adrenal Δ5 androgens also reduce cellular immunity and autoantibody production [25,26,27,28,30], support the recovery of Th1-dominated cytokine response, and their 7α/β,16α-hydroxy-metabolites counteract the weakening of the primary immune response [31].

In summary, low pregnenolone levels may reduce protection against demyelination in MS patients, while higher levels of its sulfate could help prevent cognitive deficits and mitigate MS-related complications. Additionally, increased levels of 7α-hydroxy-DHEA and androstenediol sulfate likely play a role in counteracting autoimmunity and inflammation.

#### 3.2.2. 11β-. Hydroxy-Androstanes (C19 Δ^4^ and 5α/β Steroids)

Our earlier study found that 11β-hydroxy-androstane levels remained mostly unchanged in female MS patients when compared with matched controls, except for 11β-hydroxy-androsterone, possibly due to the small sample size and limited statistical power [32]. However, current findings suggest reduced CYP11B1 functioning in MS patients compared to controls, as four 11β-hydroxy-androstanes exhibited significantly lower levels in MS patients, one showed no change between the groups, and none had higher levels. Despite the generally lower levels of 11β-hydroxy-androstanes, patients had higher levels of immunosuppressive cortisol and its inactive form, cortisone.

#### 3.2.3. GABAergic Steroids

Steroids associated with GABAergic effects showed significantly lower levels of 5α/β-steroids and 3α-hydroxy-5α/β-steroids in patients. These findings align with previous results, which revealed lower levels of the neuroprotective allopregnanolone and a higher ratio of antagonistic 3β-hydroxy-steroids to their neuroprotective 3α-hydroxy counterparts compared to controls [32].

#### 3.2.4. 17-oxo- and 17 β-Hydroxy-Androstanes

17-oxo-androstanes tended to have lower levels in patients, while 17β-hydroxy-androstanes did not. This may suggest reduced HSD17B2 functioning and/or increased AKR1C3 functioning in peripheral tissues. These findings align with previous results showing a borderline trend of higher ratios of 17β-hydroxy-steroids to their 17-oxo counterparts in MS patients [32].

AKR1C3 is highly expressed in immune cells, the adrenal zona reticularis, and various other tissues [33,34] http://biogps.org/#goto=genereport&id=8644, accessed on 18 February 2025. CNS inflammation and immune dysfunction are linked to MS development [1,35]. Besides its role in steroid production, AKR1C3 acts as prostaglandin (PG) F2α synthase, with PGF2α and its active metabolite 8-iso-PGF2α promoting oxidative stress and inflammation [36]. These findings suggest that increased peripheral AKR1C3 functioning may contribute to higher inflammatory responses in MS patients.

### 3.3. Correlation Between Indices of MS Severity and Steroids

Significant correlations were found between MS severity indices and steroids in both follicular and luteal phases, emphasizing the critical role of CSF steroids in MS pathophysiology.

The indices of MS severity inversely correlate with the neuroprotective pregnenolone, neuroprotective and immunomodulatory DHEA/S, and also with the immunomodulatory androstenediol and DHEA and androstenediol immunomodulatory 7α/β- and 16α-hydroxy- metabolites. In addition, the indices of MS severity inversely correlated with several GABAergic and potentially GABAergic steroids with a hydroxy-group in the 3α-position. Taken together, these data suggest a positive role for the above protective steroids in preventing MS progression.

MS severity indices are inversely associated with neuroprotective pregnenolone, immunomodulatory and neuroprotective DHEA/S, as well as androstenediol and its 7α/β- and 16α-hydroxy-metabolites. Additionally, they show inverse correlations with several GABAergic and potentially GABAergic steroids containing a hydroxy group in the 3α-position. These findings indicate that these protective steroids may play a beneficial role in preventing MS progression.

Positive correlations between T25-FW in FP and conjugated 5α/β-steroids (Table 5) were consistently observed, including those with a hydroxy group in the 3α-position, which might act as positive modulators of GABA_A_R after their desulfation by steroid sulfatase. In their conjugated form, these steroids may be either inactive, or some—such as sulfated 5α-pregnanes, which positively modulate excitatory NMDA receptors, and sulfated isopregnanolone, which negatively modulates inhibitory GABA_A_R—may even counteract the effects of their free counterparts (see review [37]).

Beyond the correlations of MS severity indices with neuroprotective and immunomodulatory steroids, additional associations suggest disruptions in the metabolic pathway to immunosuppressive cortisol, such as inverse correlations with 17- and 16α-hydroxy-pregnanes. This may indicate a link between MS severity and reduced CYP17A1 functioning in the hydroxylase step, which is primarily active in the adrenal zona fasciculata but negligible in the CNS for both its hydroxylase and lyase functions. In contrast, the lyase step of CYP17A1 is mainly active in the adrenal zona reticularis, and the observed inverse correlation between CSF androstanes and androgen production in this region may reflect declining zona reticularis function as MS progresses (see [19,38] and http://biogps.org/#goto=genereport&id=1586, accessed on 18 February 2025).

With the exception of certain positive correlations between some 11β-hydroxy-androstanes and T25-FW in FP, these steroids generally showed negative correlations with other MS severity indices. This suggests that reduced CYP11B1 functioning, which is essential for the final step of cortisol synthesis, may play a role in MS disease progression.

## 4. Potential Clinical Implications of the Findings

Building on the findings outlined above, these insights carry profound clinical implications, shedding light on potential avenues for therapeutic intervention and disease management. The observed alterations in steroid levels in MS patients suggest disruptions in steroidogenesis and metabolism, which may contribute to disease progression and symptom severity. Specifically, reduced neuroprotective and immunomodulatory steroids indicate a potential target for therapeutic intervention. Enhancing the levels of pregnenolone, DHEA, and their sulfate forms, as well as modulating enzymes like CYP11B1 and AKR1C3, could help mitigate inflammation and neurodegeneration associated with MS. Additionally, the role of sulfotransferase functioning and altered cortisol metabolism highlights the need for further investigation into hormonal regulation in MS. These insights may pave the way for novel treatment strategies aimed at restoring steroid balance and improving patient outcomes.

## 5. Future Directions

Looking ahead, these findings pave the way for several promising research directions. Further investigation into the mechanisms underlying steroid metabolism in MS could lead to novel biomarkers for disease progression and treatment response. Additionally, exploring therapeutic strategies aimed at modulating key enzymes such as CYP11B1 and AKR1C3 may offer new avenues for intervention. Future studies should also examine the potential benefits of restoring neuroprotective and immunomodulatory steroids to mitigate inflammation and neurodegeneration. A deeper understanding of the interplay between peripheral and central steroidogenesis could refine approaches to personalized medicine, ultimately improving patient outcomes.

## 6. Limitations and Strengths of the Study

Compared to our previous steroidomic studies, which evaluated changes in the steroidome in circulation in female MS patients versus controls, as well as the impact of anti-MS treatment on the steroidome, the current study primarily focuses on assessing steroidomic changes in CSF in female patients compared to controls across both phases of the menstrual cycle. Due to the significantly lower levels of endogenous steroids in CSF compared to circulation, the number of analyzed steroids was lower, which may have somewhat limited the interpretative value of the obtained results. On the other hand, steroidomic changes in CSF could more accurately reflect the situation in the CNS compared to circulation, in terms of the role of steroidomic alterations in the pathophysiology of MS. Compared to our previous study, which evaluated changes in the steroidome in circulation in female MS patients versus the control group, in the current study, we had a larger number of observed volunteers, which should lead to an increase in statistical testing power. Moreover, unlike the previous study, we now examined changes in both phases of the menstrual cycle, whereas the previous study was limited to the follicular phase only.

## 7. Materials and Methods

### 7.1. Subjects

The study included 74 women with MS aged 37.7 (29.1, 42.5) years (shown as median with quartiles) and 40 female controls 37.8 (32.0, 44.1) years. Among the MS patients, 57 were in the FP and 17 in the LP, while the controls included 24 in FP and 16 in LP. MS diagnoses were confirmed through CSF analysis and MRI, and all patients met the 2017 McDonald criteria [39]. The MS patients were newly diagnosed and untreated, and those with a history of COVID were excluded. The study was approved by the Ethics Committee of the General University Hospital, Prague, Czech Republic (Approval number: 74/19, 20 June 2019). For steroidome evaluation, CSF was collected using a sterile atraumatic needle, centrifuged, and stored at −20 °C until analysis.

### 7.2. Steroid Analysis

Steroids and their polar conjugates in the cerebrospinal fluid (CSF) were measured using our slightly modified GC-MS/MS method [40]. The modifications included (1) reducing the number of internal standards due to observed stability issues with [2,2,4,6,21-D8]-17α-hydroxyprogesterone and [2,2,4,6,17α,21-D9]-progesterone in both serum and CSF analysis; (2) using CSF instead of serum as the sample matrix in CSF analysis; and (3) replacing charcoal-stripped serum with chromatography-grade water for the preparation of calibration samples in CSF analysis.

In brief, after addition of the mixed stock solution of internal standards such as [2,2,3,4,4,6-D6]-DHEA, [9,11,12,12-D4]cortisol and [2,2,4,6,6,12,12-D7]-cortisone, into the CSF sample and mixing (1 min), the unconjugated steroids were extracted from 1 mL of this mixture with diethyl-ether (3 mL). The diethyl-ether extract was dried in a block heater at 37 °C. The lipids in the dry residue of the diethyl-ether extract were separated by partitioning between a mixture of methanol with water 4:1 (1 mL) and pentane (1 mL). The pentane phase was discarded and the polar phase was dried in a vacuum centrifuge at 60 °C (2 h). The dry residue from the polar phase was firstly dissolved in 100 μL of acetonitrile. The solution was transferred into the 1 mL conical vial and dried in the flow of nitrogen. The dry residue was derivatized first with a methoxyamine hydrochloride solution in pyridine (2%) (60 °C, 1 h) to convert the oxo-groups to methyloxime derivatives. After this first derivatization, the mixture was dried in a flow of nitrogen and the dry residue was treated with the reagent Sylon BTZ (90 °C, 24 h). The Sylon BTZ is a mixture of N,O-bis(trimethylsilyl)acetamide (BSA) + trimethylchlorosilane (TMCS) + N-trimethylsilylimidazole (TMSI) (3:2:3). After this second derivatization step, the mixture was dried in the nitrogen flow (2 min). After administration of approximately 1 mg of ammonium bicarbonate, the residue was partitioned between isooctane (100 μL) and N,N-dimethylformamide (50 μL). Then the volume of the vial was mixed (1 min) and centrifuged for 20 min at 3000 rpm. The lower, polar layer was aspirated with a Pasteur pipette and the upper non-polar layer remained in the vial for GC-MS/MS analysis. From the upper layer, 2 μL was injected into the GC-MS/MS system.

Steroid conjugates remaining in the polar residue after diethyl ether extractions were analyzed as follows: The volume of 15 μL D6-DHEA sulfate solution (50 μg/mL) was mixed with this residue (1 min mixing). Then 1 mL of methanol was added and mixed for an additional 1 min. After the centrifugation of the mixture (20 min at 3000 rpm), the upper layer was transferred to the clean 10 mL extraction tube, dried in the vacuum centrifuge at 37 °C (5 h), and the dry residues were chemically hydrolyzed according to Dehennin and Peres [41]. Briefly, 1 mL of 1 M TMCS was added to the dry residue of the upper layer and after 1 min mixing, the hydrolysis proceeded for 1 h at 55 °C. Then 100 mg of sodium bicarbonate was added and after short mixing, the hydrolyzed samples were again dried in the vacuum centrifuge at 37 °C (5 h). The dried residues were reconstituted with 500 μL of chromatographic water and then further processed in the same way as the free steroids.

The instrument used was a GCMS-TQ8040 system from Shimadzu (Kyoto, Japan) consisting of a gas chromatograph equipped with an automatic flow control, an AOC-20s autosampler and a triple quadrupole detector with an adjustable electron voltage of 10–195 V. The analysis was conducted in multiple reaction monitoring (MRM) mode. A capillary column with a medium polarity RESTEK Rtx-50 column (diameter 0.25 mm, length 15 m, film thickness 0.1 μm) was used for analyses. Electron-impact ionization with electron voltage fixed at 60 V and emission current set to 151 μA was used for the measurements. The temperatures of the injection port, ion source and interface were maintained at 220, 300, and 310 °C, respectively. Analyses were carried out in the splitless mode with a constant linear velocity of the carrier gas (He), which was maintained at 60 cm/s. The septum purge flow was set to 3 mL/min. The samples were injected using a high-pressure mode, which was applied at 200 kPa and maintained for 1 min. The detector voltage was set to 2.2 kV. The temperature program was as follows: 1 min delay at 80 °C, increase to 190 °C (40 °C/min), increase to 210 °C (6 °C/min), increase to 300 °C (20 °C/min), increase to 320 °C (40 °C/min), 4 min delay at 320 °C, initial pressure 34 kPa, injector temperature 220 °C, analysis duration 16.08 min.

The calibration was performed in chromatography-grade water. The analytes were quantified using calibration curves based on known concentrations in the mixtures of analyzed standards with constant levels of ISs. We used a 9-point logarithmic calibration curve. The values were corrected for procedural losses according to the yields of ISs. The amount of each steroid injected from the calibration samples into the GC corresponded to amounts of 10 ng, 2 ng, 500 pg, 125 pg, 31.2 pg, 7.81 pg, 1.95 pg, 488 fg and 122 fg. The calibration curves were constructed by plotting the logarithm of the response factor (analyte area/internal standard area) against the logarithm of the concentration of the calibration (external) standard to cover the large concentration differences for circulating steroids in different physiological and pathophysiological situations and even more explicit contrasts between unconjugated steroids and their conjugated counterparts at an appropriate number of calibration points.

### 7.3. Statistical Analysis

Power transformations were applied to each metric variable to approximate a Gaussian distribution. Data were analyzed using ANOVA and orthogonal projections to latent structure (OPLS) models, with the latter focusing on differences between patients and controls. An age-adjusted ANCOVA model was used to account for age-related effects, incorporating MS status (patients vs. controls), menstrual cycle phase (FP vs. LP), and their interaction (MS × PMC).

Statgraphics Centurion v. XVIII was used for power transformations and ANOVA analysis, while SIMCA-P v.12.0 handled OPLS analysis. The OPLS models assessed differences between patients and controls and explored relationships between MS severity parameters and the steroidome. Details on the OPLS method were provided in a previous study [32]. In addition to the aforementioned study, this study also shows CV-ANOVA functionality that evaluates the model by testing whether it provides significant predictive capabilities.

When interpreting the data, we did not assess individual steroids but rather all steroids or their groups. The final trend was analyzed using the one-sample Wilcoxon test, incorporating reductions in patients compared to controls (value = −1), insignificant changes (value = 0), and increases in patients compared to controls (value = 1). These approaches helped minimize the risk that an individual statistically significant false positive result would influence the interpretation of findings.

## 8. Conclusions

The key findings from the analysis of steroid levels in CSF of untreated female MS patients compared to controls are as follows:

(1) Both unconjugated and conjugated steroids exhibited a strong correlation between the circulation and CSF, suggesting steroid transfer from the bloodstream to the CNS and underscoring the significance of peripheral steroidogenesis.
(2) The noticeable decrease in unconjugated steroid levels implies diminished steroidogenesis in MS patients compared to controls. In contrast, the absence of significant changes in conjugated steroids could indicate heightened functioning of sulfotransferase (SULT2A1) in these patients.
(3) Reduced functioning of adrenal 11β-hydroxylase (CYP11B1), essential for the final step of cortisol synthesis, has been observed in MS patients. Additionally, impaired cortisol metabolism, involving decreased CYP17A1 and CYP11B1 functioning, was associated with more severe MS.
(4) Reduced levels of 5α/β-steroids and protective GABAergic 3α-hydroxy-5α/β-steroids in MS patients might be linked to the pathophysiology of MS.
(5) The steroidomic data indicates that higher AKR1C3 functioning in MS patients might cause inflammation, as this enzyme is involved in the production of both steroids and prostaglandins.
(6) Reduced pregnenolone levels in MS patients could weaken protection against demyelination, whereas elevated pregnenolone sulfate levels in this group might help safeguard against cognitive deficits.
(7) MS severity was inversely associated with neuroprotective pregnenolone, its sulfate, DHEA, its sulfate, and immunomodulatory steroids such as androstenediol and its hydroxy-metabolites, highlighting their potentially protective role in MS.

## Figures and Tables

**Table 1:** Pearson’s correlations (r) between steroid levels in serum and cerebrospinal fluid (data after power transformations to attain symmetry and constant variance in the distribution of individual variables).

Steroid	r	*p*-Value		Steroid	r	*p*-Value
Pregnenolone	0.303	0.002		5α,20α-Tetrahydroprogesterone	0.499	<0.001
Pregnenolone sulphate	0.315	<0.001		Conjugated 5α-pregnane-3α,20α-diol	0.492	<0.001
17-Hydroxypregnenolone	0.641	<0.001		Conjugated 5α-pregnane-3β,20α-diol	0.727	<0.001
16α-Hydroxypregnenolone	0.632	<0.001		Conjugated 5β-pregnane-3α,20α-diol	0.428	<0.001
20α-Dihydropregnenolone sulphate	0.473	<0.001		Conjugated 5β-pregnane-3β,20α-diol	0.61	<0.001
Dehydroepiandrosterone (DHEA)	0.479	<0.001		5α-Pregnane-3α,17,20α-triol	0.499	<0.001
DHEA sulphate	0.692	<0.001		5β-Pregnane-3α,17,20α-triol	0.723	<0.001
7α-Hydroxy-DHEA	0.63	<0.001		Androsterone	0.477	<0.001
7β-Hydroxy-DHEA	0.749	<0.001		Androsterone sulphate	0.488	<0.001
Androstenediol	0.28	0.003		Epiandrosterone sulphate	0.38	<0.001
Androstenediol sulphate	0.561	<0.001		Etiocholanolone sulphate	0.453	<0.001
5-Androstene-3β,7α,17β-triol	0.662	<0.001		Epietiocholanolone sulphate	0.5	<0.001
5-Androstene-3β,7β,17β-triol	0.567	<0.001		Conjugated 5α-androstane-3α,17β-diol	0.512	<0.001
5-Androstene-3β,16α,17β-triol sulphate	0.644	<0.001		Conjugated 5α-androstane-3β,17β-diol	0.698	<0.001
17,20α-Dihydroxy-4-pregnene-3-one	0.418	<0.001		Conjugated 5β-androstane-3α,17β-diol	0.33	<0.001
16α-Hydroxyprogesterone	0.801	<0.001		11β-Hydroxyandrostenedione	0.205	0.058
Androstenedione	0.412	<0.001		11β-Hydroxyandrosterone	0.771	<0.001
Conjugated pregnanolone	0.688	<0.001		11β-Hydroxyetiocholanolone sulphate	0.878	<0.001

**Table 2:** Steroid levels in the cerebrospinal fluid of female patients with multiple sclerosis (MS+) and in aged-matched controls (MS−) in follicular (FP) and luteal (LP) phases.

					ANOVA, *p*-Values					Trend MS+, (*p* < 0.05)					
Steroid	Follicular, MS−	Follicular, MS+	Luteal, MS−	Luteal, MS+	MS	PMC	MS×PMC	Age		Factor MS	MC, FP	OPLS, FP	MC, LP	OPLS, LP	Trend
Pregnenolone [pM]	32.9 (26, 41.7)	19.4 (16.7, 22.5)	17.5 (13.1, 23.4)	15.7 (12, 20.5)	0.066	0.016	0.224	0.380			↓				**↓**
Pregnenolone sulphate [pM]	94.3 (74, 121)	108 (91.1, 128)	80.6 (60.6, 108)	220 (154, 321)	0.004	0.177	0.029	0.009		↑			↑		**↑**
17-Hydroxypregnenolone [pM]	31 (23.1, 41.7)	42.2 (34.7, 51.3)	41.6 (29.2, 59.4)	27.3 (19.6, 37.9)	0.787	0.741	0.091	0.401							
16α-Hydroxypregnenolone [pM]	6.41 (4.81, 8.55)	3.1 (2.61, 3.68)	4.07 (2.98, 5.57)	2.3 (1.69, 3.11)	0.001	0.057	0.693	0.193		↓	↓	↓		↓	**↓**
20α-Dihydropregnenolone sulphate [pM]	320 (259, 395)	350 (304, 402)	402 (308, 525)	450 (348, 583)	0.533	0.137	0.941	0.658							
Dehydroepiandrosterone (DHEA) [pM]	143 (111, 184)	117 (98.7, 138)	111 (81.5, 151)	108 (80, 146)	0.539	0.383	0.642	0.154							
DHEA sulphate [nM]	0.571 (0.454, 0.715)	0.634 (0.545, 0.736)	0.82 (0.624, 1.08)	1.25 (0.93, 1.69)	0.130	0.003	0.351	0.829							
7α-Hydroxy-DHEA [nM]	0.328 (0.269, 0.396)	0.841 (0.766, 0.922)	0.474 (0.386, 0.577)	0.897 (0.747, 1.07)	0.000	0.092	0.263	0.009		↑	↑		↑		**↑**
7β-Hydroxy-DHEA [pM]	86.8 (66.8, 111)	81.9 (69.1, 96.3)	63.3 (45.5, 85.5)	45.6 (31.7, 63.3)	0.342	0.023	0.529	0.236							
Androstenediol [pM]	12.2 (9.71, 15.1)	10.3 (8.76, 12.1)	12.1 (9.03, 15.8)	16.1 (12.8, 20)	0.672	0.175	0.162	0.849				↓			**↓**
Androstenediol sulphate [nM]	1.23 (1.03, 1.47)	1.39 (1.23, 1.56)	1 (0.801, 1.25)	2.56 (2.06, 3.18)	0.000	0.114	0.003	0.052		↑			↑		**↑**
5-Androstene-3β,7α,17β-triol [pM]	110 (83.5, 143)	111 (93, 131)	82.3 (58.5, 114)	129 (94.5, 175)	0.249	0.741	0.267	0.428							
5-Androstene-3β,7β,17β-triol [pM]	35.5 (27.9, 45.2)	32.1 (27.3, 37.8)	32.4 (24, 43.7)	34.5 (25.8, 46)	0.920	0.955	0.657	0.060							
5-Androstene-3β,16α,17β-triol sulphate [pM]	72.1 (57.3, 90.4)	31.4 (26.8, 36.6)	45.4 (33.7, 60.6)	23 (16.8, 31)	0.000	0.032	0.604	0.007		↓	↓		↓		**↓**
17,20α-Dihydroxy-4-pregnene-3-one [pM]	11.8 (9.52, 14.6)	15.2 (13.2, 17.6)	41.6 (32.7, 52.7)	16.6 (12.7, 21.4)	0.024	0.000	0.000	0.048		↓			↓	↓	**↓**
16α-Hydroxyprogesterone [pM]	9.45 (6.83, 12.9)	15.3 (12.6, 18.5)	60.1 (43.8, 82.1)	48.9 (35.5, 66.9)	0.575	0.000	0.111	0.017						↓	**↓**
Androstenedione [pM]	75.5 (65, 87.4)	73.4 (67.1, 80.2)	83 (70.9, 96.7)	111 (94.8, 129)	0.182	0.012	0.109	0.000				↓			**↓**
Conjugated pregnanolone [pM]	63.5 (52.4, 76.2)	68.5 (59.9, 78)	143 (118, 171)	137 (112, 167)	0.930	0.000	0.661	0.238							
5α,20α-Tetrahydroprogesterone [pM]	3.62 (2.76, 4.77)	2.73 (2.33, 3.21)	9.08 (6.59, 12.7)	6.56 (4.87, 8.95)	0.118	0.000	0.974	0.047						↓	**↓**
Conjugated 5α-pregnane-3α,20α-diol [pM]	162 (133, 198)	96.1 (84.7, 109)	393 (296, 525)	330 (260, 422)	0.025	0.000	0.236	0.092		↓	↓				**↓**
Conjugated 5α-pregnane-3β,20α-diol [pM]	66.5 (42.3, 106)	36.6 (26.7, 50.1)	91.5 (52.3, 164)	323 (176, 620)	0.409	0.001	0.011	0.044					↑		**↑**
Conjugated 5β-pregnane-3α,20α-diol [pM]	54.3 (43.6, 67.2)	73.5 (63.5, 85)	168 (130, 219)	222 (171, 289)	0.077	0.000	0.951	0.010							
Conjugated 5β-pregnane-3β,20α-diol [pM]	53.7 (45.5, 63.3)	63.3 (56.6, 70.7)	105 (85.8, 128)	78.2 (63.9, 95.8)	0.621	0.001	0.068	0.049							
5α-Pregnane-3α,17,20α-triol [pM]	0.534 (0.391, 0.74)	0.38 (0.312, 0.465)	0.634 (0.424, 0.974)	0.502 (0.354, 0.726)	0.222	0.337	0.804	0.006				↓		↓	**↓**
5β-Pregnane-3α,17,20α-triol [pM]	18.1 (14.9, 21.8)	25.6 (22.8, 28.7)	39.5 (33, 46.9)	22.8 (18.5, 28)	0.343	0.008	0.001	0.167			↑		↓		
Androsterone [pM]	29.5 (25, 34.9)	18.8 (17, 20.7)	47.8 (39.2, 58.8)	6.73 (5.38, 8.37)	0.000	0.015	0.000	0.071		↓	↓		↓	↓	**↓**
Androsterone sulphate [pM]	460 (381, 559)	307 (271, 347)	363 (292, 455)	292 (236, 363)	0.025	0.307	0.512	0.005		↓	↓				**↓**
Epiandrosterone sulphate [pM]	151 (119, 189)	180 (155, 208)	170 (127, 225)	158 (116, 211)	0.775	0.982	0.473	0.324							
Etiocholanolone sulphate [pM]	132 (112, 155)	126 (113, 140)	126 (104, 153)	116 (94, 144)	0.620	0.617	0.889	0.520				↓			**↓**
Epietiocholanolone sulphate [pM]	47.3 (38.3, 58.1)	60.9 (52.6, 70.4)	42.3 (32.8, 54.3)	41.8 (31.7, 54.4)	0.443	0.125	0.395	0.857				↓			**↓**
Conjugated 5α-androstane-3α,17β-diol [pM]	532 (422, 673)	405 (349, 472)	333 (252, 441)	337 (255, 447)	0.459	0.062	0.420	0.069				↑			**↑**
Conjugated 5α-androstane-3β,17β-diol [pM]	143 (112, 182)	108 (91.1, 128)	106 (77.4, 142)	108 (78.3, 146)	0.481	0.402	0.419	0.845							
Conjugated 5β-androstane-3α,17β-diol [pM]	18.9 (15.2, 23.4)	20.3 (17.5, 23.5)	30.9 (23.4, 40.7)	23.7 (18.2, 30.7)	0.557	0.052	0.299	0.645							
11β-Hydroxyandrostenedione [nM]	2.09 (1.87, 2.33)	1.82 (1.69, 1.96)	2.35 (2.07, 2.65)	1.9 (1.67, 2.15)	0.030	0.321	0.614	0.087		↓					**↓**
11β-Hydroxyandrosterone [pM]	182 (147, 225)	91.1 (77.2, 107)	131 (98.9, 171)	82.4 (61.3, 109)	0.001	0.196	0.459	0.001		↓	↓	↓		↓	**↓**
11β-Hydroxyandrosterone sulphate [pM]	116 (105, 128)	110 (102, 119)	137 (119, 157)	93.6 (82, 106)	0.009	0.890	0.044	0.881		↓			↓		**↓**
11β-Hydroxyetiocholanolone [pM]	75.4 (61.2, 91.8)	66.9 (58.1, 76.8)	84.4 (67.8, 104)	62.5 (49.3, 78.2)	0.143	0.864	0.522	0.001				↓		↓	**↓**
11β-Hydroxyetiocholanolone sulphate [pM]	149 (120, 182)	191 (166, 218)	233 (186, 289)	193 (152, 242)	0.860	0.115	0.137	0.917							

PMC = phase of menstrual cycle, MS × PMC = interaction of multiple sclerosis with PMC, ↑ = higher in MS+ (patients) compared with MS− (controls), ↓ = lower in MS+ compared with MS−.

## Data Availability

Data available on request due to restrictions (i.e., privacy, legal and ethical reasons).

## Supplementary Materials

The following supporting information can be downloaded at: https://www.mdpi.com/article/10.3390/ijms26125904/s1.

## References

[1] Ysrraelit M.C., Correale J. (2019). Impact of sex hormones on immune function and multiple sclerosis development. Immunology.

[2] Sparaco M., Bonavita S. (2021). The role of sex hormones in women with multiple sclerosis: From puberty to assisted reproductive techniques. Front. Neuroendocr..

[3] Ubuka T., Trudeau V.L., Parhar I. (2020). Editorial: Steroids and the Brain. Front. Endocrinol..

[4] Redzic Z. (2011). Molecular biology of the blood-brain and the blood-cerebrospinal fluid barriers: Similarities and differences. Fluids Barriers CNS.

[5] Porcu P., Barron A.M., Frye C.A., Walf A.A., Yang S.Y., He X.Y., Morrow A.L., Panzica G.C., Melcangi R.C. (2016). Neurosteroidogenesis Today: Novel Targets for Neuroactive Steroid Synthesis and Action and Their Relevance for Translational Research. J. Neuroendocr..

[6] Duque Ede A., Munhoz C.D. (2016). The Pro-inflammatory Effects of Glucocorticoids in the Brain. Front. Endocrinol..

[7] Borowicz K.K., Piskorska B., Banach M., Czuczwar S.J. (2011). Neuroprotective actions of neurosteroids. Front. Endocrinol..

[8] Giatti S., Boraso M., Melcangi R.C., Viviani B. (2012). Neuroactive steroids, their metabolites, and neuroinflammation. J. Mol. Endocrinol..

[9] Melcangi R.C., Giatti S., Pesaresi M., Calabrese D., Mitro N., Caruso D., Garcia-Segura L.M. (2011). Role of neuroactive steroids in the peripheral nervous system. Front. Endocrinol..

[10] De Alcubierre D., Ferrari D., Mauro G., Isidori A.M., Tomlinson J.W., Pofi R. (2023). Glucocorticoids and cognitive function: A walkthrough in endogenous and exogenous alterations. J. Endocrinol. Investig..

[11] Gundamraj S., Hasbun R. (2020). The Use of Adjunctive Steroids in Central Nervous Infections. Front. Cell. Infect. Microbiol..

[12] Ngo S.T., Steyn F.J., McCombe P.A. (2014). Gender differences in autoimmune disease. Front. Neuroendocr..

[13] Smith R., Studd J.W. (1992). A pilot study of the effect upon multiple sclerosis of the menopause, hormone replacement therapy and the menstrual cycle. J. R. Soc. Med..

[14] Argyriou A.A., Makris N. (2008). Multiple sclerosis and reproductive risks in women. Reprod. Sci..

[15] Begemann M.J., Dekker C.F., van Lunenburg M., Sommer I.E. (2012). Estrogen augmentation in schizophrenia: A quantitative review of current evidence. Schizophr. Res..

[16] Qaiser M.Z., Dolman D.E.M., Begley D.J., Abbott N.J., Cazacu-Davidescu M., Corol D.I., Fry J.P. (2017). Uptake and metabolism of sulphated steroids by the blood-brain barrier in the adult male rat. J. Neurochem..

[17] Powrie Y.S.L., Smith C. (2018). Central intracrine DHEA synthesis in ageing-related neuroinflammation and neurodegeneration: Therapeutic potential?. J. Neuroinflamm..

[18] Honcu P., Hill M., Bicikova M., Jandova D., Velikova M., Kajzar J., Kolatorova L., Bestak J., Macova L., Kancheva R. (2019). Activation of Adrenal Steroidogenesis and an Improvement of Mood Balance in Postmenopausal Females after Spa Treatment Based on Physical Activity. Int. J. Mol. Sci..

[19] Kamin H.S., Kertes D.A. (2017). Cortisol and DHEA in development and psychopathology. Horm. Behav..

[20] Noorbakhsh F., Baker G.B., Power C. (2014). Allopregnanolone and neuroinflammation: A focus on multiple sclerosis. Front. Cell. Neurosci..

[21] Balan I., Beattie M.C., O’Buckley T.K., Aurelian L., Morrow A.L. (2019). Endogenous Neurosteroid (3alpha,5alpha)3-Hydroxypregnan-20-one Inhibits Toll-like-4 Receptor Activation and Pro-inflammatory Signaling in Macrophages and Brain. Sci. Rep..

[22] Kancheva R., Hill M., Novak Z., Chrastina J., Velikova M., Kancheva L., Riha I., Starka L. (2010). Peripheral neuroactive steroids may be as good as the steroids in the cerebrospinal fluid for the diagnostics of CNS disturbances. J. Steroid Biochem. Mol. Biol..

[23] Bottasso O., Bay M.L., Besedovsky H., del Rey A. (2007). The immuno-endocrine component in the pathogenesis of tuberculosis. Scand. J. Immunol..

[24] Du C., Khalil M.W., Sriram S. (2001). Administration of dehydroepiandrosterone suppresses experimental allergic encephalomyelitis in SJL/J mice. J. Immunol..

[25] Rontzsch A., Thoss K., Petrow P.K., Henzgen S., Brauer R. (2004). Amelioration of murine antigen-induced arthritis by dehydroepiandrosterone (DHEA). Inflamm. Res..

[26] Tan X.D., Dou Y.C., Shi C.W., Duan R.S., Sun R.P. (2009). Administration of dehydroepiandrosterone ameliorates experimental autoimmune neuritis in Lewis rats. J. Neuroimmunol..

[27] Choi I.S., Cui Y., Koh Y.A., Lee H.C., Cho Y.B., Won Y.H. (2008). Effects of dehydroepiandrosterone on Th2 cytokine production in peripheral blood mononuclear cells from asthmatics. Korean J. Intern. Med..

[28] Sudo N., Yu X.N., Kubo C. (2001). Dehydroepiandrosterone attenuates the spontaneous elevation of serum IgE level in NC/Nga mice. Immunol. Lett..

[29] Romagnani S., Kapsenberg M., Radbruch A., Adorini L. (1998). Th1 and Th2 cells. Res. Immunol..

[30] Pratschke S., von Dossow-Hanfstingl V., Dietz J., Schneider C.P., Tufman A., Albertsmeier M., Winter H., Angele M.K. (2014). Dehydroepiandrosterone modulates T-cell response after major abdominal surgery. J. Surg. Res..

[31] Sterzl I., Hampl R., Sterzl J., Votruba J., Starka L. (1999). 7Beta-OH-DHEA counteracts dexamethasone induced suppression of primary immune response in murine spleenocytes. J. Steroid Biochem. Mol. Biol..

[32] Kancheva R., Hill M., Velikova M., Kancheva L., Vcelak J., Ampapa R., Zido M., Stetkarova I., Libertinova J., Vosatkova M. (2024). Altered Steroidome in Women with Multiple Sclerosis. Int. J. Mol. Sci..

[33] Nakamura Y., Hornsby P.J., Casson P., Morimoto R., Satoh F., Xing Y., Kennedy M.R., Sasano H., Rainey W.E. (2009). Type 5 17beta-hydroxysteroid dehydrogenase (AKR1C3) contributes to testosterone production in the adrenal reticularis. J. Clin. Endocrinol. Metab..

[34] Luu-The V. (2013). Assessment of steroidogenesis and steroidogenic enzyme functions. J. Steroid Biochem. Mol. Biol..

[35] Murgia F., Giagnoni F., Lorefice L., Caria P., Dettori T., D’Alterio M.N., Angioni S., Hendren A.J., Caboni P., Pibiri M. (2022). Sex Hormones as Key Modulators of the Immune Response in Multiple Sclerosis: A Review. Biomedicines.

[36] Xu C., Liu W., You X., Leimert K., Popowycz K., Fang X., Wood S.L., Slater D.M., Sun Q., Gu H. (2015). PGF2alpha modulates the output of chemokines and pro-inflammatory cytokines in myometrial cells from term pregnant women through divergent signaling pathways. Mol. Hum. Reprod..

[37] Vitku J., Hill M., Kolatorova L., Kubala Havrdova E., Kancheva R. (2022). Steroid Sulfation in Neurodegenerative Diseases. Front. Mol. Biosci..

[38] Singh H., Kumar R., Mazumder A., Salahuddin, Mazumder R., Abdullah M.M. (2022). Insights into Interactions of Human Cytochrome P450 17A1: A Review. Curr. Drug Metab..

[39] Thompson A.J., Banwell B.L., Barkhof F., Carroll W.M., Coetzee T., Comi G., Correale J., Fazekas F., Filippi M., Freedman M.S. (2018). Diagnosis of multiple sclerosis: 2017 revisions of the McDonald criteria. Lancet Neurol..

[40] Hill M., Hana V., Velikova M., Parizek A., Kolatorova L., Vitku J., Skodova T., Simkova M., Simjak P., Kancheva R. (2019). A method for determination of one hundred endogenous steroids in human serum by gas chromatography-tandem mass spectrometry. Physiol. Res..

[41] Dehennin L., Peres G. (1996). Plasma and urinary markers of oral testosterone misuse by healthy men in presence of masking epitestosterone administration. Int. J. Sports Med..

