# Supplementary material for: Steroidomic Changes in the Cerebrospinal Fluid of Women with Multiple Sclerosis
Source: Int J Mol Sci. 2025 Jun 19;26(12):5904. doi: 10.3390/ijms26125904 (PMC12193344; doi:10.3390/ijms26125904)
Supplement: Supplementary file 1 [file ijms-26-05904-s001.zip › Figure S1, Table S1.pdf]

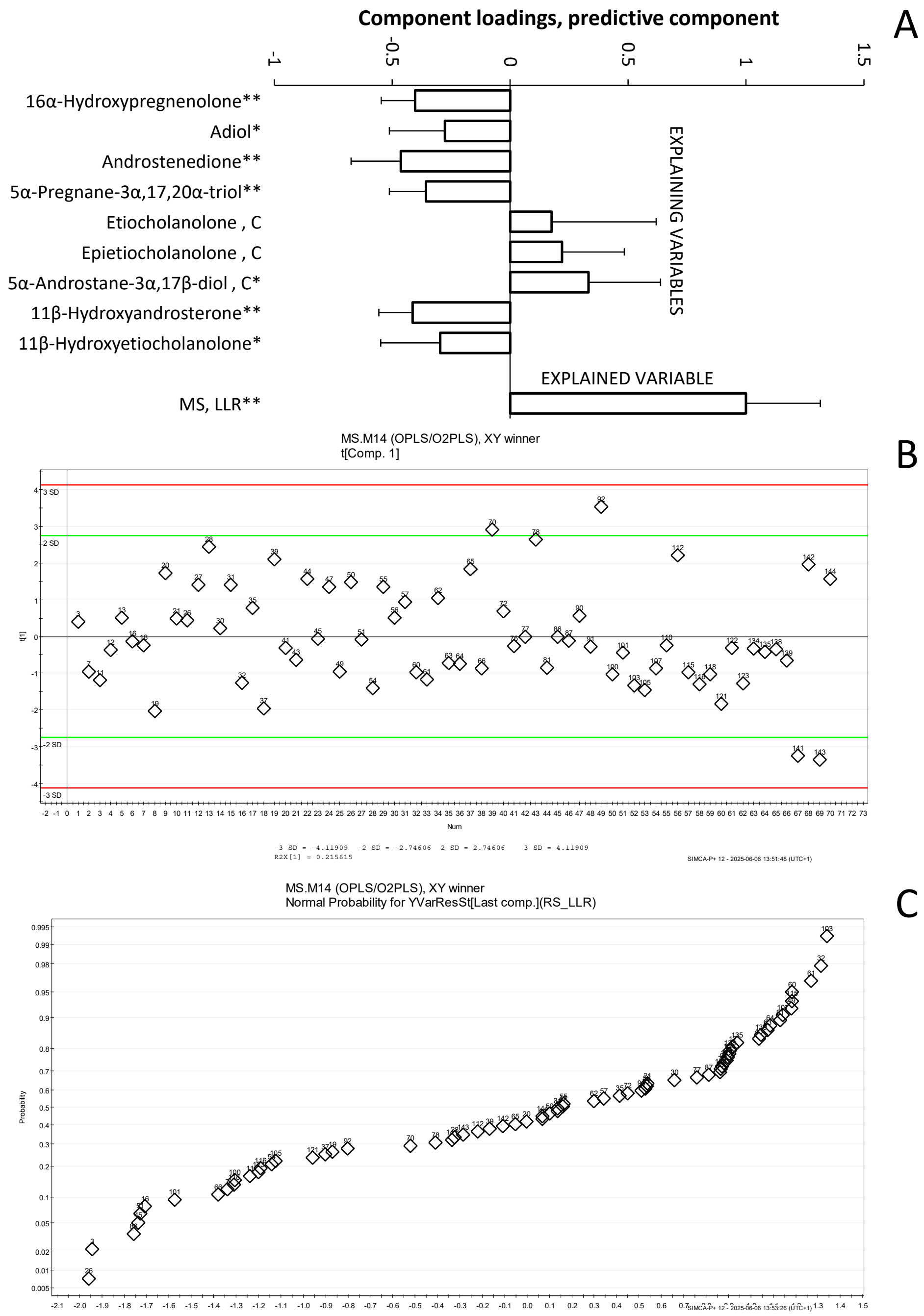

Figure S1. Discrimination between groups of patients with MS and controls based on steroids as evaluated by models of orthogonal predictions to latent structure (OPLS) and ordinary multiple regression (OMR) for follicular menstrual phase. Diagnostic outputs for OPLS, Panel A: Component loadings for predictive component, Panel B: Score plot, *p<0.05, **p<0.01, Panel C: Residual plot. The image complements Table S1.
