# Supplementary material for: Steroidomic Changes in the Cerebrospinal Fluid of Women with Multiple Sclerosis
Source: Int J Mol Sci. 2025 Jun 19;26(12):5904. doi: 10.3390/ijms26125904 (PMC12193344; doi:10.3390/ijms26125904)
Supplement: Supplementary file 1 [file ijms-26-05904-s001.zip › Figure S2, Table S2.pdf]

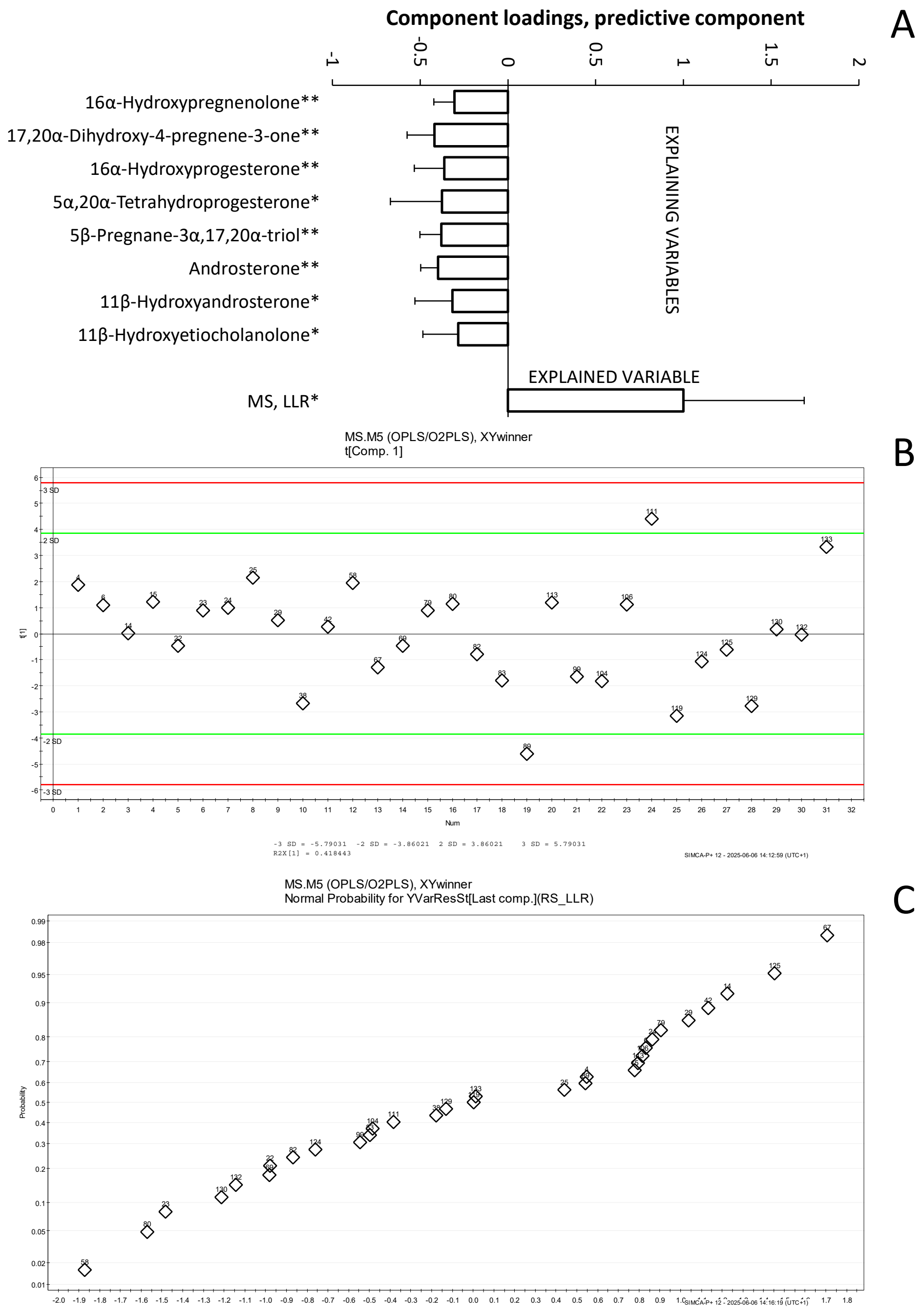

Figure S2. Discrimination between groups of patients with MS and controls based on steroids as evaluated by models of orthogonal predictions to latent structure (OPLS) and ordinary multiple regression (OMR) for luteal menstrual phase. Diagnostic outputs for OPLS, Panel A: Component loadings for predictive component *p<0.05, **p<0.01, Panel B: Score plot, Panel C: Residual plot. The image complements Table S2.
