# Supplementary material for: Steroidomic Changes in the Cerebrospinal Fluid of Women with Multiple Sclerosis
Source: Int J Mol Sci. 2025 Jun 19;26(12):5904. doi: 10.3390/ijms26125904 (PMC12193344; doi:10.3390/ijms26125904)
Supplement: Supplementary file 1 [file ijms-26-05904-s001.zip › Figure S5, Table 5.pdf]

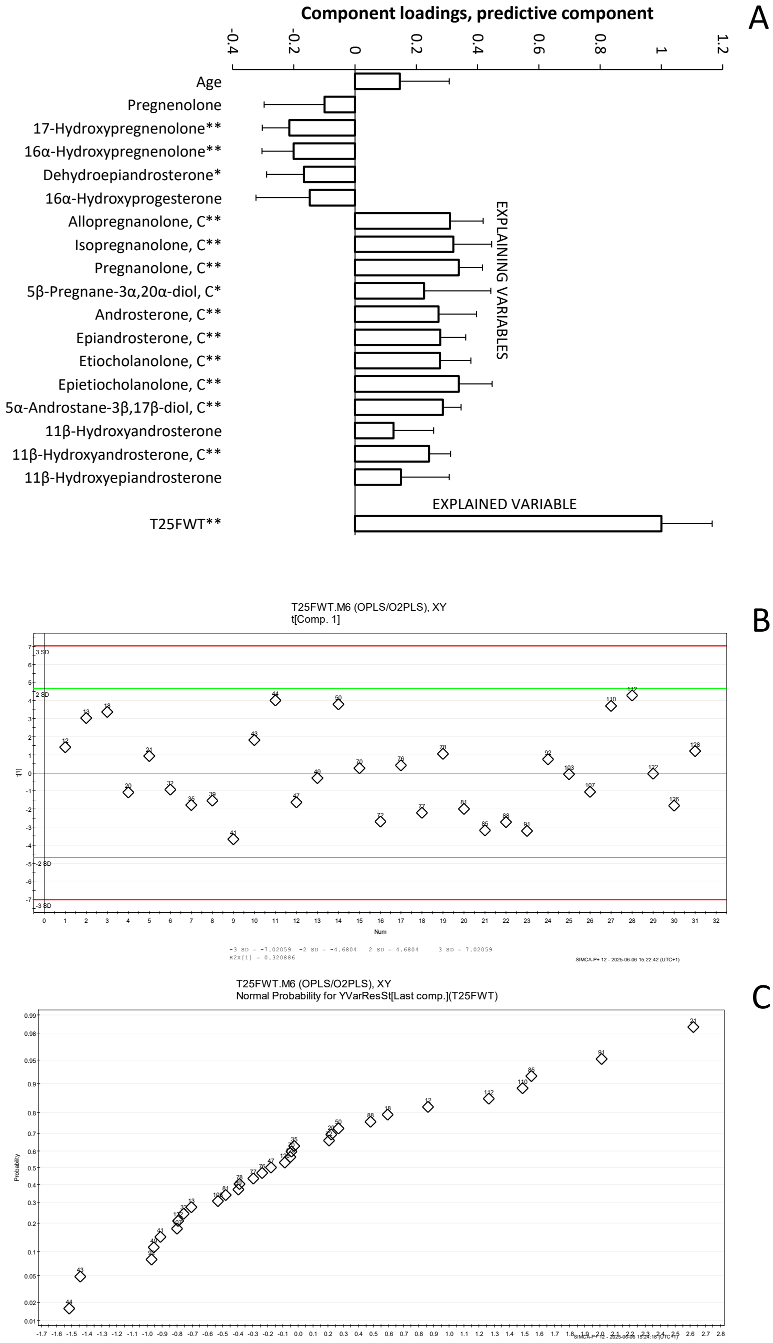

Figure S5. Relationships between T25FWT and explaining variables as evaluated by models of orthogonal predictions to latent structure (OPLS) and ordinary multiple regression (OMR) for follicular menstrual phase Diagnostic outputs for OPLS, Panel A: Component loadings for predictive component, *p<0.05, **p<0.01, Panel B: Score plot, Panel C: Residual plot. The image complements Table 5.
