# Supplementary material for: Steroidomic Changes in the Cerebrospinal Fluid of Women with Multiple Sclerosis
Source: Int J Mol Sci. 2025 Jun 19;26(12):5904. doi: 10.3390/ijms26125904 (PMC12193344; doi:10.3390/ijms26125904)
Supplement: Supplementary file 1 [file ijms-26-05904-s001.zip › Table S2, CSF steroids patients vs. controls, LP, OPLS.pdf]

Table S2. Discrimination between groups of patients with MS and controls based on steroids in the luteal phase as evaluated by models of orthogonal predictions to latent structure (OPLS) and ordinary multiple regression (OMR). Corresponding diagnostic outputs are shown in supplementary Figure S2.

		**OPLS,** predictive component					**Multiple regression**
	**Variable**	Variable importance	t-statistic	Component loading	t-statistic	R	t-statistic
EXPLAINING VARIABLES	16α-Hydroxypregnenolone	0.995	2.33*	-0.305	-4.93	-0.560**	-2.47*
	17,20α-Dihydroxy-4-pregnene-3-one	0.922	2.63*	-0.418	-5.09	-0.768**	-1.74
	16α-Hydroxyprogesterone	0.972	3.20**	-0.362	-4.01	-0.666**	-2.91*
	5α,20α-Tetrahydroprogesterone	0.944	2.07*	-0.375	-2.41	-0.689*	-2.09*
	5β-Pregnane-3α,17,20α-triol	0.999	3.17**	-0.379	-5.87	-0.696**	-2.26*
	Androsterone	1.261	3.26**	-0.398	-7.71	-0.732**	-2.68*
	11β-Hydroxyandrosterone	1.106	3.10**	-0.316	-2.79	-0.580*	-2.14*
	11β-Hydroxyetiocholanolone	0.718	2.53*	-0.284	-2.68	-0.521*	-2.63*
EXPLAINED VARIABLE	Multiple sclerosis, LLR			1.000	2.75	0.495*	
	Luteal phase	R^2=24.5%, Q^2=16.4%, CV-ANOVA: F=2.95, p=0.068					

*R=Component loading expressed as a correlation coefficient with predictive component, *p<0.05, **p<0.01, R^2=Explained variance, Q^2=Predictive ability, CV-ANOVA=cross validated ANOVA, F=F-statistic, p=statistical significance*
